# Cross-modality network phenotyping: a multivariate MRI biomarker signature predicts spatial pathology and clinical severity in EAE

**DOI:** 10.3389/fneur.2026.1850801

**Published:** 2026-09-17

**Authors:** Rohan S. Virgincar, Man Kin Choy, Surinder Jeet, Hunter Martinez, Luca Ghita, Ronal Peralta, Hannah Bender, Joshua D. Webster, Kai Barck, Hye-Jung Kim, Subba Chintalacharuvu, Luke Xie

**Affiliations:** 1 Department of Translational Imaging, Genentech, South San Francisco, CA, United States; 2 Department of Translational Immunology, Genentech, South San Francisco, CA, United States; 3 Department of Immunology, Genentech, South San Francisco, CA, United States; 4 Department of Research Pathology, Genentech, South San Francisco, CA, United States

**Keywords:** biomarkers, diffusion tensor imaging (DTI), EAE (experimental autoimmune encephalomyelitis), magnetic resonance imaging (MRI), multiple sclerosis (MS), neuroinflammation, spinal cord

## Abstract

**Introduction:**

Evaluating novel therapies for multiple sclerosis requires robust, non-invasive biomarkers. In the experimental autoimmune encephalomyelitis (EAE) model, standard disease evaluations rely heavily on subjective clinical motor scoring and terminal histology, which frequently suffer from a mathematical dilution effect. To address this, we developed an automated, spatially targeted multiparametric magnetic resonance imaging (MRI) pipeline to extract highly specific *in vivo* biophysical markers and benchmarked its performance against a potent positive control immunomodulator, fingolimod.

**Methods:**

EAE was induced in mice using a myelin oligodendrocyte glycoprotein peptide, followed by treatment with either vehicle or fingolimod. At 28 days post-induction, *in vivo* T2-weighted and diffusion tensor imaging maps of the lumbar spinal cord were acquired and segmented into specific gray and white matter columns. The extracted MRI parameters were cross-correlated with clinical scores, spatial histopathology, and flow cytometry.

**Results:**

Vehicle-treated mice exhibited severe elevations in gray matter T2 relaxation times and precipitous drops in white matter fractional anisotropy, structurally mapping to localized edema, demyelination, and dense cellular infiltrates. Fingolimod treatment successfully rescued these structural deficits, alongside clinical motor deficits and T-cell infiltration. Localized metrics, specifically ventral white matter fractional anisotropy and a composite fractional anisotropy to T2 ratio, demonstrated treatment effect sizes that exceeded those of the gross clinical paralysis score and corresponding lumbar histopathology. Multivariate predictive modeling showed that the combined MRI panel predicted up to 87.2% of the variance in regional spinal cord pathology and 80.1% of the variance in clinical paralysis. Furthermore, unsupervised principal component analysis extracted an MRI disease score that strongly correlated with clinical disability, global histopathology, and total central nervous system T-cells.

**Conclusion:**

Spatially targeted, multiparametric MRI serves as a highly sensitive, objective surrogate biomarker signature. By overcoming the dilution effect, this non-invasive approach provides an objective, continuous metric that complements traditional tissue assays, enhancing the evaluation of multiple sclerosis therapeutics while advancing the reduction of animal use.

## Introduction

Multiple sclerosis (MS) is a complex, demyelinating, and neurodegenerative disease of the central nervous system (CNS). While current disease-modifying therapies effectively target peripheral immunomodulation to reduce relapse rates, there remains an urgent clinical need to develop and validate novel neuroprotective, deeper immune-dampening, and remyelinating agents to address compartmentalized CNS inflammation (1–3). To rigorously evaluate these experimental therapeutics, the myelin oligodendrocyte glycoprotein (MOG35-55) experimental autoimmune encephalomyelitis (EAE) mouse model remains the preclinical standard. As a classically T-cell-mediated model, EAE faithfully recapitulates the hallmark of focal neuroinflammation, demyelination, and escalating motor paralysis seen in severe MS (4–6).

However, evaluating disease severity and therapeutic efficacy in the EAE model relies heavily on macroscopic clinical motor scoring and terminal *ex vivo* histopathology. While historically foundational, these standard methodologies harbor significant limitations. Clinical motor scoring (typically a 0–5 scale) is inherently subjective, suffers from inter-rater variability, and cannot mechanistically distinguish between paralysis driven by transient vasogenic edema versus irreversible axonal transection (7, 8). Furthermore, *ex vivo* histology provides only a static, terminal snapshot of a dynamic disease process. To construct a temporal profile of drug efficacy, researchers are forced to use large, independent animal cohorts across staggered time points, inflating biological variance and substantially increasing the logistical burden of preclinical studies. This approach limits experimental throughput and runs counter to the 3Rs (Replacement, Reduction, Refinement) framework (9).

Additionally, traditional global or whole-section tissue evaluations frequently suffer from a mathematical “dilution effect”. Because EAE pathology is highly multifocal, mathematically averaging microscopic, severe lesions with surrounding healthy tissue often masks nuanced, localized tissue destruction, resulting in poor correlations with actual functional disability (the clinicoradiological paradox) (10).

To bridge this preclinical-to-clinical translational gap, multiparametric magnetic resonance imaging (MRI) has emerged as a critical non-invasive tool (11–14). By utilizing quantitative T2 relaxation mapping to assess edema and inflammatory fluid shifts, alongside Diffusion Tensor Imaging (DTI) to probe the microstructural, directional integrity of axons and myelin (Fractional Anisotropy, FA), researchers can extract highly specific *in vivo* biophysical markers. Crucially, the non-invasive nature of MRI enables true longitudinal, repeated-measures study designs. This allows individual animals to serve as their own internal baseline controls, drastically reducing the required sample sizes while increasing statistical power. Furthermore, by isolating specific microstructural tracts, targeted MRI parameters offer a robust, continuous mathematical scale that provides critical spatial context to complement traditional invasive tissue scoring.

In this study, we present an automated, high-resolution multiparametric MRI pipeline designed to combat the dilution effect. This approach maps microstructural damage to functionally relevant spatial locations, specifically targeting the ventral white matter motor tracts of the lumbar spinal cord. To definitively benchmark the biological and spatial fidelity of this non-invasive platform, we evaluated its performance against a known, highly potent positive control immunomodulator, fingolimod (FTY720). We hypothesize that this spatially targeted, cross-modality MRI signature will accurately predict gold-standard clinical and pathological outcomes. Specifically, we anticipate that localized structural imaging will yield treatment effect sizes that not only rival but exceed standard *ex vivo* regional pathology assays. Ultimately, this approach serves as a highly sensitive, non-invasive surrogate to enhance the evaluation of future MS therapeutics.

## Materials and methods

### Animals and ethics statement

All animal activities, including procedures, anesthesia, and euthanasia methods, were conducted in accordance with the Guide for the Care and Use of Laboratory Animals (National Research Council 2011), and all animals were maintained in a facility accredited by AAALAC International. All animal activities in this research study were reviewed and approved by the Genentech Institutional Animal Care and Use Committee (IACUC) and were performed in accordance with all institutional program requirements. All euthanasia was performed following IACUC approved methods that also follow American Veterinary Medical Association (AVMA) Guidelines for the Euthanasia of Animals.

Female C57BL/6 J mice (JAX stock #000664; The Jackson Laboratory), aged 8–9 weeks at the start of the study, were housed in a specific pathogen-free facility under standard conditions with a 12-h light/dark cycle and ad libitum access to food and water. To strictly control for confounding biological and environmental variables standard in translational research, cohorts were genetically identical, age-matched, and housed uniformly within the same facility room. Animals were randomized into experimental groups based on baseline body weight prior to disease induction.

### Experimental autoimmune encephalomyelitis (EAE) induction and treatment

To induce T-cell-dependent EAE, mice were immunized using a myelin oligodendrocyte glycoprotein MOG35-55 peptide emulsion kit (Hooke Laboratories). Mice were anesthetized using isoflurane (2.5% in pure O_2_) and subcutaneously injected with a total of 200 μL of MOG35-55 emulsion in Complete Freund’s Adjuvant (CFA). The CFA was supplemented with heat-killed *Mycobacterium tuberculosis* (H37Ra) at a concentration of 1 mg/mL (resulting in a total dose of 200 μg of *Mycobacterium tuberculosis* per animal). The emulsion was divided into two 100 μL injections administered to the midline of the upper back and the lower back. To facilitate blood–brain barrier permeabilization and disease onset, mice received an intraperitoneal (i.p.) injection of pertussis toxin (110 ng per mouse in 100 μL of PBS) one hour after the MOG immunization on Day 0, followed by a second identical pertussis toxin injection on Day 1. A cohort of healthy, non-immunized C57BL/6 J mice was maintained in parallel as a naïve control group.

Therapeutic intervention commenced on Day 7 post-immunization, prior to the onset of peak motor symptoms. Diseased mice were assigned to receive either Fingolimod Hydrochloride (FTY720; 3 mg/kg) or a 100% phosphate-buffered saline (PBS) vehicle control. Both fingolimod and the PBS vehicle were administered via intraperitoneal injection (100 μL volume) three times per week (e.g., Monday, Wednesday, Friday) for the duration of the study.

A total of 25 mice were enrolled in the *in vivo* longitudinal tracking portion of the study. At study termination on Day 28, the *in vivo* cohorts were randomly allocated to either fixed-tissue or fresh-tissue processing to accommodate the incompatible tissue requirements of histopathology and flow cytometry. A comprehensive breakdown of animal sample sizes per experimental endpoint is provided in Table 1.

**Table 1:** Animal sample sizes per experimental endpoint.

Study phase	Endpoint/Assay	Naïve (Control)	Vehicle (EAE)	FTY720 (EAE)	Total (N)
*In Vivo* phenotyping	Clinical Scoring	5	10	10	25
	Body Weight	5	10	10	25
	Multiparametric MRI^a^	5	9	9	23
*Ex Vivo* (fixed tissue)^b^	Histopathology (H&E, LFB)	2	5	5	12
	Immunohistochemistry (Iba1)	2	5	5	12
*Ex Vivo* (fresh tissue)^b^	Flow Cytometry (T-cells)	3	5	5	13

^a^Two animals (one Vehicle, one FTY720) were excluded from the final MRI analyses due to severe scan artifacts. ^b^At study termination (Day 28), the surviving cohorts were randomly allocated to either fixed-tissue pathways (perfusion/formalin fixation for Histology and IHC) or fresh-tissue pathways (tissue dissociation for Flow Cytometry), resulting in a split sample size for *ex vivo* assays.

### Clinical evaluation of EAE

Animals were monitored for clinical signs of EAE and weighed at least three times per week by a trained investigator. This longitudinal measurement framework was maintained continuously from baseline through study termination to rigorously track animal development and physical presentation across all cohorts. Starting on Day 7 post-immunization, the frequency of clinical monitoring was increased to daily or twice daily if animals exhibited severe weight loss (>20%) or advanced disease symptoms. Clinical scoring was conducted continuously until the study termination at Day 28 post-immunization. Clinical disability was graded using a highly stratified 0–5 scoring system to accurately capture subtle motor deficits, adapted from widely implemented EAE behavioral grading protocols (4, 8):

- 0: Normal; no overt signs of disease.
- 0.5: Partial limp tail (partial flaccidity of the tail with preserved curling at the tip).
- 1.0: Complete limp tail (complete flaccidity with no curling at the tip when lifted).
- 1.5: Partial hindlimb weakness (manifested as a waddling gait).
- 2.0: Hindlimb weakness (hindlimbs fall through the bars when the mouse is placed on a wire cage top).
- 3.0: Partial hindlimb paresis (mouse cannot maintain rump posture or walk normally but retains some movement in both hindlimbs).
- 3.5: Partial hindlimb paralysis (complete paralysis of one hindlimb with residual movement in the other).
- 4.0: Complete hindlimb paralysis (total loss of movement in both hindlimbs; mouse drags itself entirely using forelimbs).
- 4.5: Complete hindlimb paralysis accompanied by moderate-to-severe forelimb weakness (animals required twice-daily supportive care, including subcutaneous fluids and accessible dietary gel).
- 5.0: Moribund or completely paralyzed in all limbs, rendering the animal unable to independently access food or water. Animals reaching humane endpoints in approved protocols were euthanized immediately.

### *In vivo* multiparametric MRI acquisition

MRI was performed at 28 days post-induction on a Bruker BioSpin 7.0 T small animal MRI scanner (ParaVision 7.0.0) utilizing quantitative T2 and DTI acquisition parameters based on established high-resolution spinal cord neuroimaging frameworks (15–17). Animals were anesthetized using isoflurane (1.5–2.0% in medical air) and vital signs (respiration and temperature) were continuously monitored and maintained throughout the scan. Due to the severe local presentation of lesions characteristic of the EAE model and the range limitations of the MRI cryoprobe, anatomical coverage for all scans was restricted to the lumbar regions of the spinal cord.

#### T2-weighted imaging (MSME)

Quantitative T2 relaxation maps were acquired using a multi-slice multi-echo (MSME) sequence. The acquisition parameters were: repetition time (TR) = 4,000 ms; 12 echoes with an initial echo time (TE) = 6.5 ms and an echo spacing of 6.5 ms; field of view (FOV) = 12 × 12 mm; matrix = 185 × 185, yielding an in-plane resolution of 65 × 65 μm; 30 slices with a slice thickness of 0.5 mm; and number of averages = 1. The total scan time was 12 min and 20 s.

#### Diffusion tensor imaging (DTI)

Diffusion-weighted images (DWI) were acquired using an echo planar based DTI sequence. Acquisition parameters were: TR = 4,000 ms; TE = 21.2 ms; FOV = 12 × 12 mm; Matrix = 96 × 96, yielding an in-plane resolution of 125 × 125 μm; 30 slices with a slice thickness of 0.5 mm; and number of averages = 1. A total of 66 DWI were acquired, consisting of 6 non-DWI (approximate b = 0 s/mm^2^) and 60 volumes with diffusion gradients applied in non-collinear directions (b = 1,000 s/mm^2^). The total scan time was 4 min and 24 s.

### MRI post-processing and parametric mapping

Image post-processing was performed in MATLAB (MathWorks, Natick, MA) and utilizing the MRtrix software package (18). To correct for hardware drift, respiratory motion, and eddy current distortions, all DWI were first co-registered to each animal’s high signal-to-noise ratio (SNR) template image using the Advanced Normalization Tools (ANTs) registration pipeline (19). Here, the template is the registration of all DWI images. Following motion correction, the b-matrix was rotated (20) and DTI parameter maps were computed to extract the apparent diffusion coefficient (ADC) and fractional anisotropy (FA). ADC represents the overall magnitude of water molecule diffusion within the voxel and is functionally proportional to Mean Diffusivity (MD). Because of this linear scalar relationship, ADC and MD capture identical statistical variance across group comparisons and correlation analyses. FA quantifies the directionality of the diffusion, ranging from 0 (completely isotropic/random diffusion) to 1 (highly restricted diffusion along a single primary axis). Quantitative T2 maps were generated by fitting the MSME echo data to a mono-exponential decay model using the open-source software package qMRLab (21). To ensure robust fitting and prevent noise-induced artifacts, the first echo (6.5 ms) was explicitly excluded from the model during the calculation of T2 relaxation times. For qualitative figure generation only, representative structural MRI and parametric maps were upscaled using bicubic interpolation; all quantitative region-of-interest extractions and statistical analyses were strictly performed at native resolution.

### Segmentation workflow

To accurately quantify region-specific neuroinflammation and demyelination, the lumbar spinal cord was segmented using the ANTs Atropos automated segmentation algorithm (22). Two separate pipelines were developed to segment the T2 and DTI acquisitions.

#### T2 segmentation

To isolate the spinal cord from surrounding background tissue, an averaged image of the final four MSME echoes (TE = 58.5–78.0 ms) was used, as these late echoes provided maximal contrast-to-noise ratio between the cord and the cerebrospinal fluid/muscle boundaries. Once the whole spinal cord mask was established, the T2 parametric map was fed into the Atropos algorithm to automatically segment the tissue into distinct gray matter (GM) and white matter (WM) regions (Figure 1).

**Figure 1:**
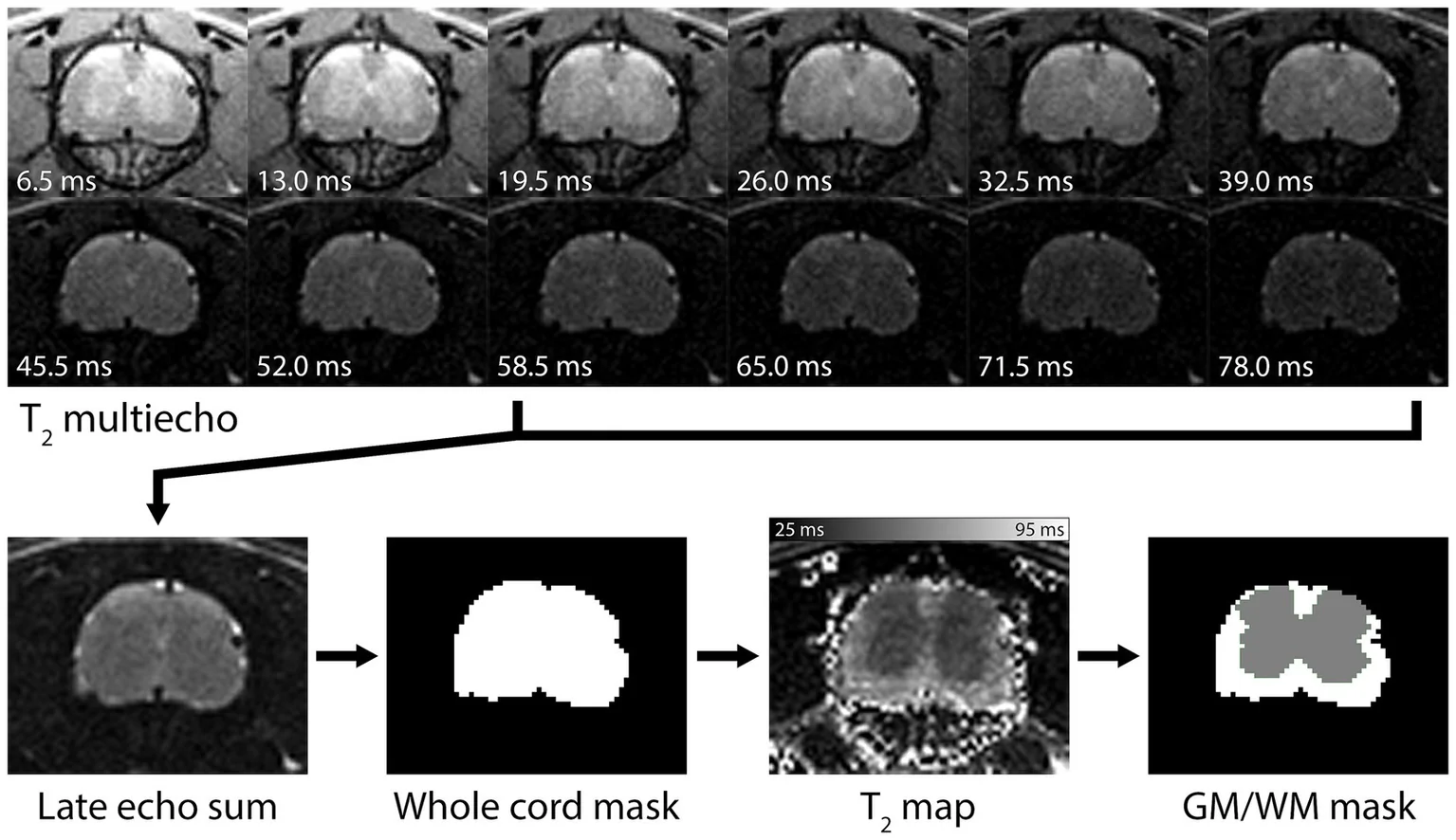
Segmentation workflow for *T2*-weighted images. To generate accurate structural masks, an average image of the last four MSME echoes was used due to its high contrast-to-noise ratio, allowing for robust isolation of the whole spinal cord. The resulting mask was applied to the quantitative *T2* map, followed by automated tissue classification using ANTs Atropos to segment the spinal cord into gray matter (GM) and white matter (WM) regions.

#### DTI segmentation

For the DTI datasets, all motion-corrected DWI were first averaged to create a high-SNR composite image, which was utilized to segment the whole spinal cord. To achieve high-fidelity segmentation of the cord, the generated boundary mask was applied exclusively to the axial diffusivity (AD) map, which was a metric chosen for its sharp GM/WM anatomical contrast allowing Atropos to segment the GM and WM regions. Finally, the isolated WM mask was split into four quadrants to yield the left lateral, right lateral, dorsal, and ventral WM columns (Figure 2). Voxel values for T2, ADC, and FA were extracted from these finalized ROI masks and averaged across the slices for each animal.

**Figure 2:**
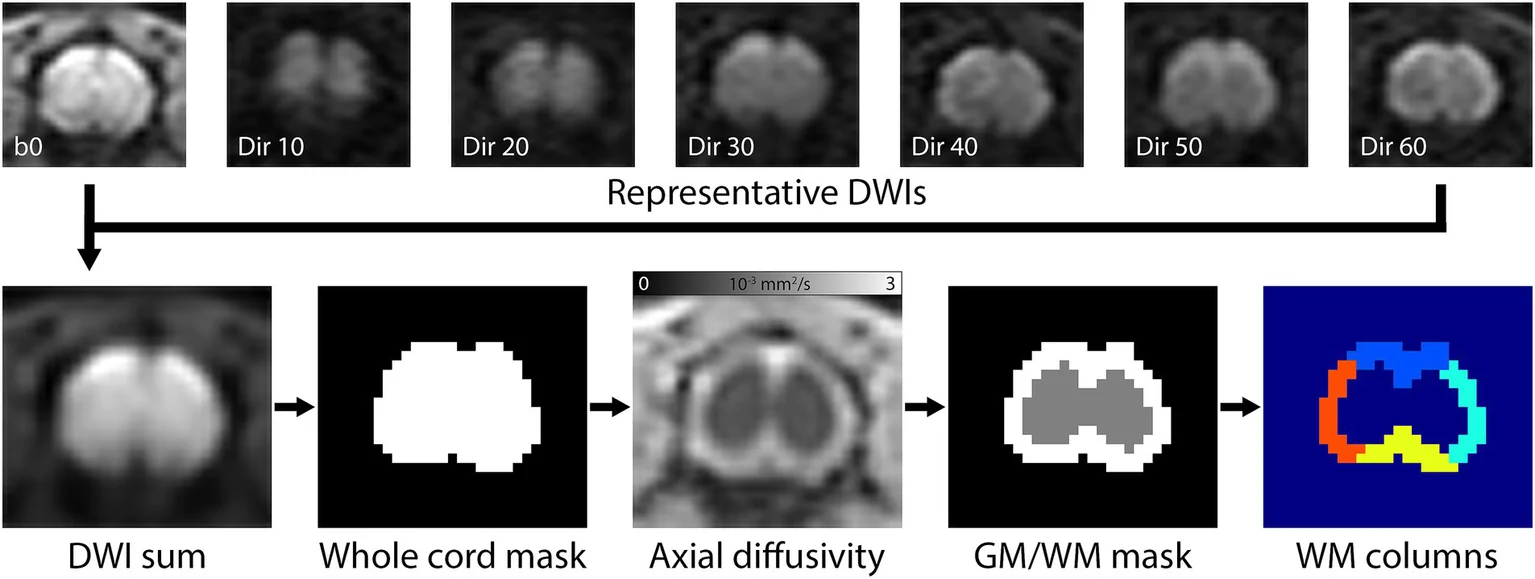
Segmentation workflow for diffusion tensor imaging (DTI). DWI volumes were averaged to create a high-contrast composite image (DWI sum), which was used to segment the whole spinal cord. This mask was subsequently applied to the axial diffusivity (AD) map to enable Atropos segmentation of the gray matter (GM) and white matter (WM). The resulting WM mask was then parcellated into the left lateral, right lateral, dorsal, and ventral WM columns for regional quantification of FA and ADC.

### Histopathology and tissue scoring

Following all final *in vivo* imaging and clinical monitoring, mice assigned to the fixed-tissue cohort were deeply anesthetized into a surgical plane of anesthesia with isoflurane (2.5% in pure O_2_) and euthanized via exsanguination by terminal transcardial perfusion with PBS while under anesthesia. Brains and intact vertebral columns (containing the spinal cord and surrounding musculature) were rapidly harvested and immediately fixed in 10% neutral-buffered formalin. Brains were fixed free-floating. To prevent mechanical damage and preserve linear neuroanatomy, spinal cords were fixed *in situ* within the vertebral column. All tissues were then embedded in paraffin. To comprehensively assess the spatial distribution of disease pathology, four cross-sections were collected from the brain, alongside four cross-sections from each of the cervical, thoracic, and lumbar spinal cord segments.

Tissue sections were stained with luxol fast blue (LFB) and hematoxylin and eosin (H&E) to simultaneously evaluate the severity of demyelination and inflammatory cellular infiltration. Slides were evaluated by a board-certified veterinary pathologist blinded to the experimental treatment groups.

A pre-established scoring matrix was utilized to grade pathological severity across different regions of the central nervous system. For the brain and spinal cord, lesions were scored on a scale of 0–3 (Table 2). The scores from all analyzed sections were averaged to generate regional sub-scores (brain, cervical, thoracic, lumbar) and a cumulative global pathology score for each animal.

**Table 2:** Histopathology scoring criteria for EAE lesions.

Score	Criteria
Brain	
0	No lesions
1	Mild, ≤3 perivascular infiltrates with no myelinopathy
2	Moderate, ≤6 perivascular infiltrates with or without extension into the neuroparenchyma and mild myelinopathy
3	Severe, >6 perivascular infiltrates or at least 1 large focus with extensive myelinopathy
Spinal cord	
0	No lesions
1	Mild, ≤3 small foci, mostly meningeal with no or only superficial myelinopathy associated with inflammation
2	Moderate, ≤5 inflammatory foci OR extensive superficial inflammation with accompanying myelinopathy
3	Severe, multifocal to locally extensive inflammation and demyelination often with extension into the GM

### Immunohistochemistry (Iba1)

To specifically assess the extent of microgliosis and macrophage infiltration, immunohistochemistry (IHC) was performed targeting ionized calcium-binding adapter molecule 1 (Iba1) on the Ventana Ultra platform (Roche Diagnostics, Tucson, AZ). Paraffin-embedded sections of the brain and spinal cord were subjected to CC1 mild antigen retrieval, blocked for non-specific binding with Ventana DISCOVERY inhibitor, and incubated with 0.25 μg/mL of primary anti-Iba1 antibody (Abcam EPR16588). Immunolabeling was detected with the Ventana Rabbit OmniMap detection system with Discovery Purple chromogen. Isotype controls were included to ensure immunolabeling specificity, and known positive brain sections were utilized as assay controls.

Iba1 immunolabeling was evaluated by a blinded, board-certified veterinary pathologist. The inflammatory burden was semi-quantitatively scored based on the quantity and distribution of Iba1-positive aggregates. These regional scores were averaged to generate the final Iba1 inflammatory scores used in the multivariate predictive modeling. The Iba1 scores are included in Table 3.

**Table 3:** Immunohistochemistry scoring criteria for Iba1.

Score	Criteria
Brain	
0	No lesions
1	Mild, ≤3 perivascular infiltrates with no myelinopathy
2	Moderate, ≤6 perivascular infiltrates with or without extension into the neuroparenchyma and mild myelinopathy
3	Severe, >6 perivascular infiltrates or at least 1 large focus with extensive myelinopathy
Spinal cord	
0	No lesions
1	Mild meningeal aggregates with minimal extension into the neuroparenchyma
2	Moderate meningeal aggregates with variable extension into the neuroparenchyma
3	Moderate meningeal aggregates with diffuse increase in Iba1-positive cells in the neuroparenchyma
4	Extensive inflammation extending into the neuroparenchyma with diffuse increase in Iba1-positive cells in the neuroparenchyma
Microglia morphology	
0	Diffuse, distribution of small microglia with fine processes
1	Diffuse distribution of small microglia with mild, multifocal regional hypertrophy
2	Diffuse distribution of small microglia with moderate, multifocal regional hypertrophy
3	Decreased distribution of glial cells, but increased regional hypertrophy

### Flow cytometry and T-cell quantification

To quantify the extent of systemic immune cell infiltration into the central nervous system, flow cytometry was performed on the dedicated fresh-tissue animal cohort at study termination (Day 28).

#### Tissue dissociation and cell isolation

Animals were deeply anesthetized into a surgical plane of anesthesia with isoflurane (2.5% in pure O_2_) and euthanized via terminal exsanguination by transcardial perfusion with cold PBS to remove circulating leukocytes. The brain and spinal cord were then rapidly harvested. Tissues were mechanically dissociated and enzymatically digested in HBSS supplemented with Collagenase D and DNase I for 30 min at 37 °C. The resulting homogenates were passed through a 100 μm cell strainer to generate single-cell suspensions. To isolate the CNS-infiltrating mononuclear cells and remove myelin debris, the suspensions were subjected to density gradient centrifugation using 40% percoll spun at 800 x g, with max acceleration and max brake.

#### Antibody staining and acquisition

Isolated mononuclear cells were washed and resuspended in FACS buffer (PBS containing 2% FBS and 0.5 mM EDTA). To exclude dead cells from the analysis, samples were first incubated with a fixable viability dye (ThermoFischer Cell Stain Kit L10119). Non-specific antibody binding was subsequently blocked by incubating the cells with an anti-CD16/32 Fc-receptor blocking antibody (CytekBio/2.4G2). For surface immunophenotyping, cells were stained with the following fluorophore-conjugated primary antibodies: anti-CD45 (BV750/30-F11), anti-CD3e (Spark Blue 550/17A2), anti-Thy1.2 (BV786/30-H12), anti-CD4 (BUV496/RM4-5), and anti-CD11b (BUV395/M1/70).

#### Gating strategy and analysis

Flow cytometry data were acquired on a Cytek Aurora 5 L and analyzed using FlowJo software (Tree Star, Ashland, OR). The gating strategy to identify the CNS-infiltrating T-cell population was strictly defined FSC-A x SSC-A/Singlets (FSC-H x FSC-A)/Live CD45+/Thy1.2 + CD3e+. To visualize the broader inflammatory landscape, the live CD45 + leukocyte parent gate was further evaluated using CD11b and CD3e to distinguish between infiltrating myeloid and lymphoid compartments. For the multivariate predictive models (PCA and cross-modality correlations), the absolute count of Total CNS T-cells (CD45+/CD3e+/Thy1.2+) was utilized as the primary cellular biomarker of neuroinflammation.

### Statistical analysis

All statistical modeling, multivariate analyses, and data visualizations were performed using MATLAB (MathWorks, Natick, MA).

Prior to inferential testing, the distribution of each continuous variable was assessed for normality using the Shapiro–Wilk test. While the majority of the continuous imaging metrics were consistent with a normal distribution ( p>0.05 ), certain variables departed from normality, including the ordinal clinical disability score and several regional white matter fractional anisotropy columns ( p≤0.05 ). To ensure analytical robustness against these distributional variations, all primary group comparisons and cross-modality correlations were additionally verified using rank-based non-parametric tests (Mann–Whitney U and Spearman’s rank correlation, respectively). Parametric and non-parametric configurations were highly concordant for all primary disease outcomes (Supplementary material). Consequently, parametric statistics are reported in the main body text to maximize interpretability, dynamic range visualization, and direct comparability with historical EAE neuroimaging literature, with univariate effect sizes evaluated via Hedges’ g .

Because animals were allocated to separate *ex vivo* processing pathways (histology vs. flow cytometry), missing data points were handled using pairwise deletion. Correlation analyses were performed strictly on available matched data pairs. Data are presented as box-and-whisker plots displaying the median and interquartile range overlaid with individual data points, unless otherwise stated. Longitudinal clinical score and body weight data are presented as the group mean ± standard error of the mean (SEM) at each time point.

#### Univariate analysis and effect size

Differences between experimental groups (Naïve, Vehicle, and FTY720) were evaluated using independent-samples *t*-tests (Welch’s *t*-test for unequal variances). The primary objective of this study was not the *de novo* discovery of pharmacological efficacy, as FTY720 is already a well-characterized, FDA-approved immunomodulator.

Given the modest, fixed cohort size, this study was designed to rank and compare the biological sensitivity of candidate MRI metrics against FTY720 rather than to detect small effects. Analytical emphasis was therefore placed on effect magnitude alongside statistical significance. Nonetheless, to ensure transparency, all primary group comparisons and network correlations are reported with both raw and multiplicity-corrected *p*-values (Bonferroni and Benjamini-Hochberg false discovery rate in Supplementary material). The principal disease-sensitive findings were robust to correction (all six primary metrics q < 0.01 by FDR; five of six significant after Bonferroni).

To quantitatively rank the sensitivity of each MRI and histological metric, effect sizes between the Vehicle and FTY720-treated EAE cohorts were calculated using Hedges’ *g*. Hedges’ *g* was selected over Cohen’s *d* to provide a more conservative and accurate estimate of effect size by applying a correction factor for small sample sizes. Because the cohort was fixed, statistical power was evaluated by a sensitivity analysis reporting the minimum detectable effect size and the achieved power at the observed effect sizes (Supplementary material); at n ≈ 10 per group the study was powered (80%, α = 0.05, two-sided) to detect effects of Hedges’ g ≳ 1.3.

#### Bivariate and network correlation analysis

Cross-modality relationships between non-invasive MRI parameters and *ex vivo* histological, cellular, or clinical outcomes were assessed using Pearson correlation coefficients (R). Pearson correlation was used because the assessed relationships were approximately linear and the majority of continuous variables were consistent with normality (Shapiro–Wilk; see Statistical Analysis); for variables departing from normality, all primary associations were confirmed with Spearman’s rank correlation, with concordant results (Supplementary material). To visualize the systemic interplay of these metrics, a correlation matrix heatmap was generated, and the holistic connectivity of the dataset was mapped using a network “Schemaball” diagram. In this network architecture, each evaluated biomarker is represented as a perimeter node, and the lines (edges) connecting them represent their linear correlations. The color and thickness of the connecting edges reflect the direction and absolute magnitude of the correlation coefficient, respectively. To quantify the global centrality of each parameter within the disease network, a cumulative node strength was calculated by summing the absolute values of all correlation coefficients ( ∑∣R∣ ) associated with that specific node.

#### Multivariate predictive modeling

To determine the combined predictive power of the structural MRI protocol, multiple linear regression models were constructed. The core MRI parameters (T2, ADC, and FA from various compartments) were utilized as continuous independent variables to predict singular pathological and clinical outcomes. Model performance and the collective spatial fidelity of the MRI metrics were evaluated via the adjusted coefficient of determination (Adjusted R^2^), which penalises model complexity.

Regression assumptions were verified prior to interpretation: linearity, normality of residuals, and homoscedasticity were confirmed by visual inspection of residual-versus-fitted and quantile-quantile plots (Supplemental material). Multicollinearity among the MRI predictors was quantified using the variance inflation factor (all <3.2; Supplementary material), indicating that the predictors contributed largely independent information.

#### Principal component analysis (PCA)

Unsupervised Principal Component Analysis (PCA) was employed to evaluate the multidimensional variance and natural phenotypic clustering of the cohorts based strictly on *in vivo* MRI data. Prior to PCA, all MRI metrics were z-score standardized to ensure equal variance weighting. Confidence ellipses (95%) were calculated to visualize group separations. The primary axis of variance, Principal Component 1 (PC1), was extracted as a composite MRI disease score. To validate the biological and translational fidelity of this non-invasive composite score, PC1 was subsequently correlated (via Pearson correlation) against three independent, cross-disciplinary pillars of disease severity: clinical score, global pathology score, and total CNS T-cell counts. Statistical significance for all applicable tests was defined as α = 0.05.

## Results

### *In vivo* MRI phenotypes correspond with histopathology

To visually validate the sensitivity of the multiparametric MRI protocol, *in vivo* T2-weighted and FA maps of the lumbar spinal cord were qualitatively assessed and compared against matched *ex vivo* histological sections (H&E, LFB, and Iba1) in Figure 3.

**Figure 3:**
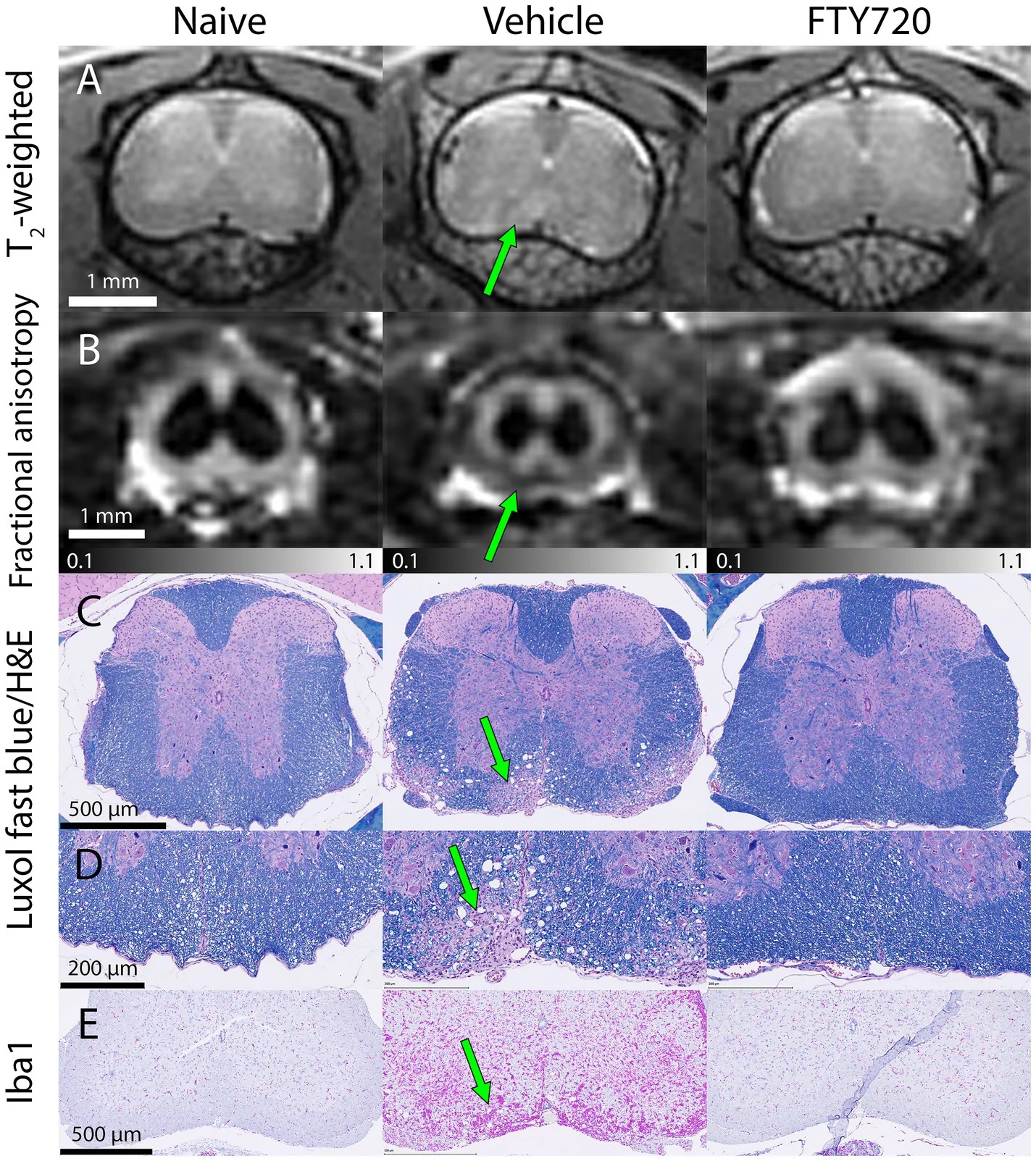
Qualitative comparison of *in vivo* MRI and *ex vivo* histopathology in the lumbar spinal cord. Representative axial T2-weighted images **(A)**, Fractional Anisotropy (FA) maps **(B)**, and corresponding histological sections [H&E and LFB in panel **(C)** and magnified in panel **(D)**; Iba1 in panel **(E)**] for naïve, vehicle, and FTY720-treated EAE mice at Day 28 post-induction. Naïve mice display distinct gray and WM boundaries and high FA signal, indicative of healthy tissue architecture and resting microglia. Vehicle-treated mice exhibit a severe loss of T2 tissue contrast and a localized reduction in FA, prominently within the ventral WM columns (green arrows). These *in vivo* radiological defects correspond directly to regions of severe, multifocal inflammation and demyelination on matched histology (green arrows). Corresponding Iba1 immunolabeling **(E)** highlights a massive accumulation of activated microglia and macrophages within these same lesion epicenters in the vehicle group. FTY720 treatment preserves structural integrity, displaying largely retained GM/WM contrast, only mild superficial myelinopathy, and heavily resolved microgliosis. For visualization purposes only, representative MRI maps were upscaled using bicubic interpolation; all quantitative analyses were performed on raw data at native resolution.

In naïve control animals, structural MRI scans displayed pristine, well-defined boundaries between the central GM and surrounding WM (Figure 3A). FA maps in these healthy animals exhibited uniformly high signal intensity across the WM columns (Figure 3B), indicative of intact, highly directional axonal tracts. This was confirmed by a complete absence of inflammatory lesions or myelinopathy on histology (Figures 3C,D), alongside normal baseline distributions of resting, ramified microglia on Iba1 staining (Figure 3E).

In stark contrast, vehicle-treated EAE mice exhibited profound macroscopic tissue disruption. T2-weighted images revealed a severe loss of GM/WM contrast, driven by diffuse edema and cellular infiltration (Figure 3A). This structural degradation was distinctly localized; lesions were most noticeable within the ventral columns of the WM. FA maps of the vehicle cohort demonstrated a severe focal decrease in directionality within these ventral tracts, suggesting a significant loss of WM integrity and axonal organization (Figure 3B). These *in vivo* radiological findings were directly corroborated by histopathology, which revealed severe, multifocal, and locally extensive inflammation and demyelination frequently extending into the GM (Figures 3C,D). Furthermore, Iba1 immunolabeling within these specific ventral lesion epicenters revealed a substantial, dense accumulation of activated microglia and infiltrating macrophages (Figure 3E).

Therapeutic intervention with the immunomodulator FTY720 visibly mitigated these pathological hallmarks. MRI scans of FTY720-treated mice retained distinct GM/WM boundaries and largely preserved the high FA signal in the ventral columns, yielding a radiological phenotype that closely resembled the naïve controls (Figures 3A,B). Corresponding histological examination confirmed this protective effect, demonstrating a marked reduction in disease burden characterized only by mild, small inflammatory foci, superficial myelinopathy, and a stark reduction in Iba1-positive microgliosis and macrophage recruitment (Figures 3C–E).

### Quantitative profiling of *in vivo* MRI parameters

Following qualitative visual confirmation, we systematically quantified the regional biophysical tissue changes across the EAE disease course and treatment paradigms (Figure 4). Quantitative MRI revealed robust, severe disease induction in the vehicle-treated EAE group compared to naïve controls. Specifically, the vehicle cohort exhibited a significant elevation in GM T2 relaxation times alongside a precipitous drop in WM FA, indicative of severe edema and demyelination, respectively (Figures 4A,F). Sub-regional parcellation of the spinal cord WM revealed that this loss of directional integrity was widespread, with FA values significantly decreased across the lateral, dorsal, and ventral columns in vehicle-treated mice (Figures 4G–J). Conversely, therapeutic intervention with FTY720 successfully rescued these structural deficits, reversing the pathological elevation in GM T2 and preventing the severe loss of WM FA across all measured columns. Notably, the composite ratio of ventral FA to GM T2 (Figure 4J) demonstrated a high dynamic range and mirrored these group-level trends, suggesting its potential utility as a highly sensitive composite imaging biomarker for localized tissue destruction (also yielded the highest MRI effect size, Figure 5).

**Figure 4:**
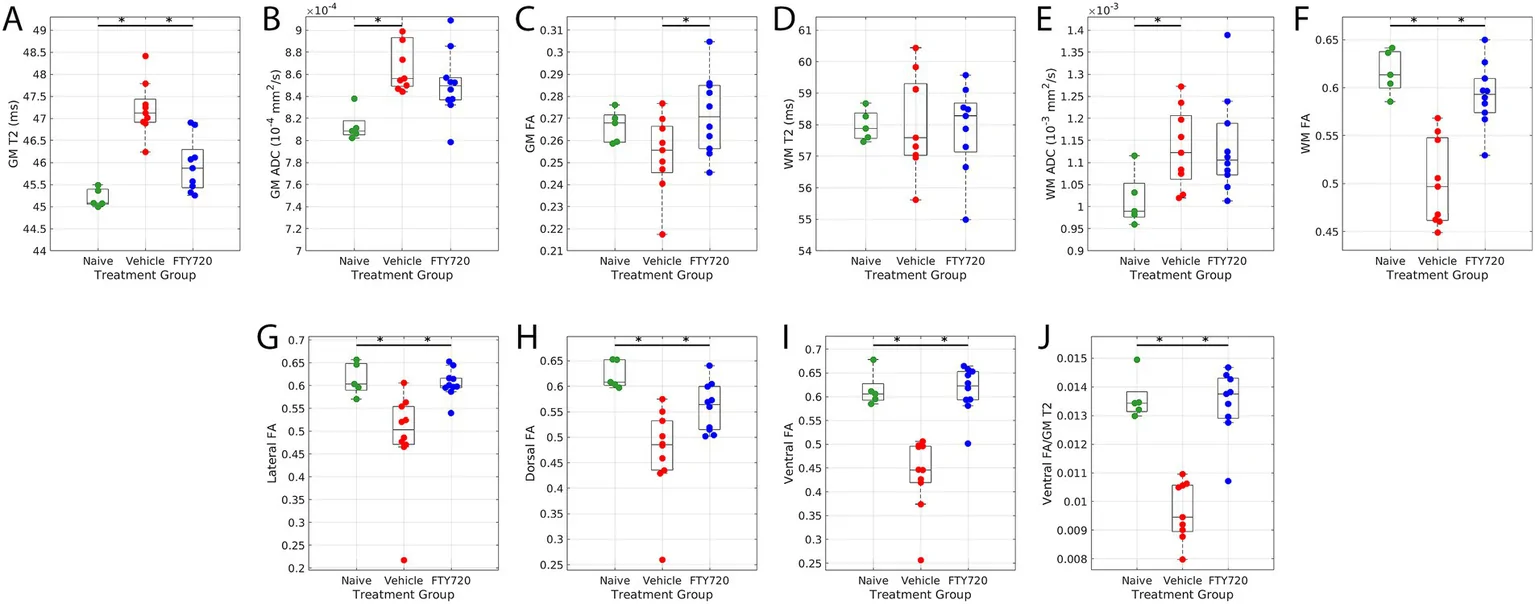
Quantitative multiparametric MRI metrics in the lumbar spinal cord. Box-and-whisker plots overlaid with individual data points demonstrating the regional biophysical changes across naïve, vehicle, and FTY720-treated EAE mice. Data are shown for the central gray matter (GM) including **(A)** T2 relaxation time, **(B)** apparent diffusion coefficient (ADC), and **(C)** fractional anisotropy (FA). Corresponding whole white matter (WM) metrics include **(D)** T2, **(E)** ADC, and **(F)** FA. Sub-regional evaluation of WM integrity demonstrates significant FA reductions in the vehicle cohort across the **(G)** lateral, **(H)** dorsal, and **(I)** ventral columns. A composite metric, the ratio of Ventral FA to GM T2 **(J)**, highlights the robust contrast between severe disease (vehicle) and preserved tissue architecture (FTY720). Boxplots display the median and interquartile range (IQR), *p < 0.05.

**Figure 5:**
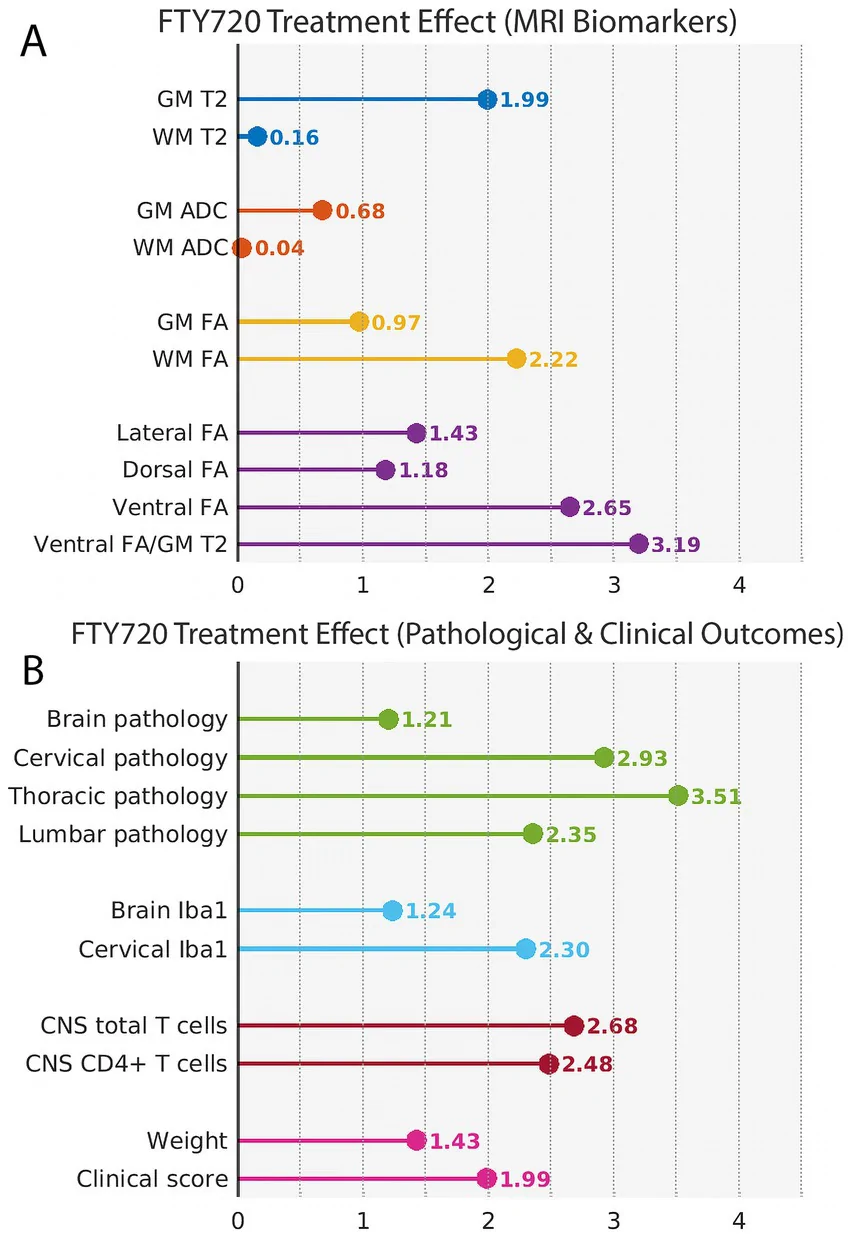
Biomarker sensitivity ranking via standardized effect sizes. Bar plots displaying the absolute effect size (Hedges’ *g*) of FTY720-mediated rescue compared to vehicle-treated EAE mice across **(A)** *in vivo* MRI parameters and **(B)** *ex vivo* biological and *in vivo* clinical endpoints. To allow for direct comparison between metrics that inherently increase (e.g., *T2*, pathology) or decrease (e.g., *FA*, body weight) following treatment, all effect sizes are plotted as absolute magnitudes. Within the MRI protocol, the ventral *FA*/*GM T2* composite ratio and localized ventral *FA* demonstrated the highest sensitivity to treatment. Biologically, spinal cord pathology scores exhibited large effects, while brain pathology showed a comparatively muted effect, consistent with the spatial distribution of the EAE model. Notably, the top-performing structural MRI metrics yielded effect sizes that met or exceeded both the gross clinical score and corresponding lumbar histopathology.

### Longitudinal clinical and systemic validation

To validate the non-invasive radiological findings, we first evaluated the systemic disease presentation across the longitudinal timeline (Figure 6). As expected, the severe structural deterioration observed on MRI in the vehicle-treated EAE cohort was closely mirrored by functional deficits and weight loss. Longitudinally, vehicle-treated mice exhibited classic disease onset post-induction, and rapidly escalated to severe clinical paralysis that persisted through study termination at day 28 (Figures 6A,B). This paralysis was accompanied by significant, sustained disease-induced weight loss (Figures 6C,D). Consistent with the structural preservation observed on MRI, therapeutic intervention with FTY720 prevented clinical motor deficits and weight loss throughout the disease course, effectively maintaining the animals near baseline naïve levels.

**Figure 6:**
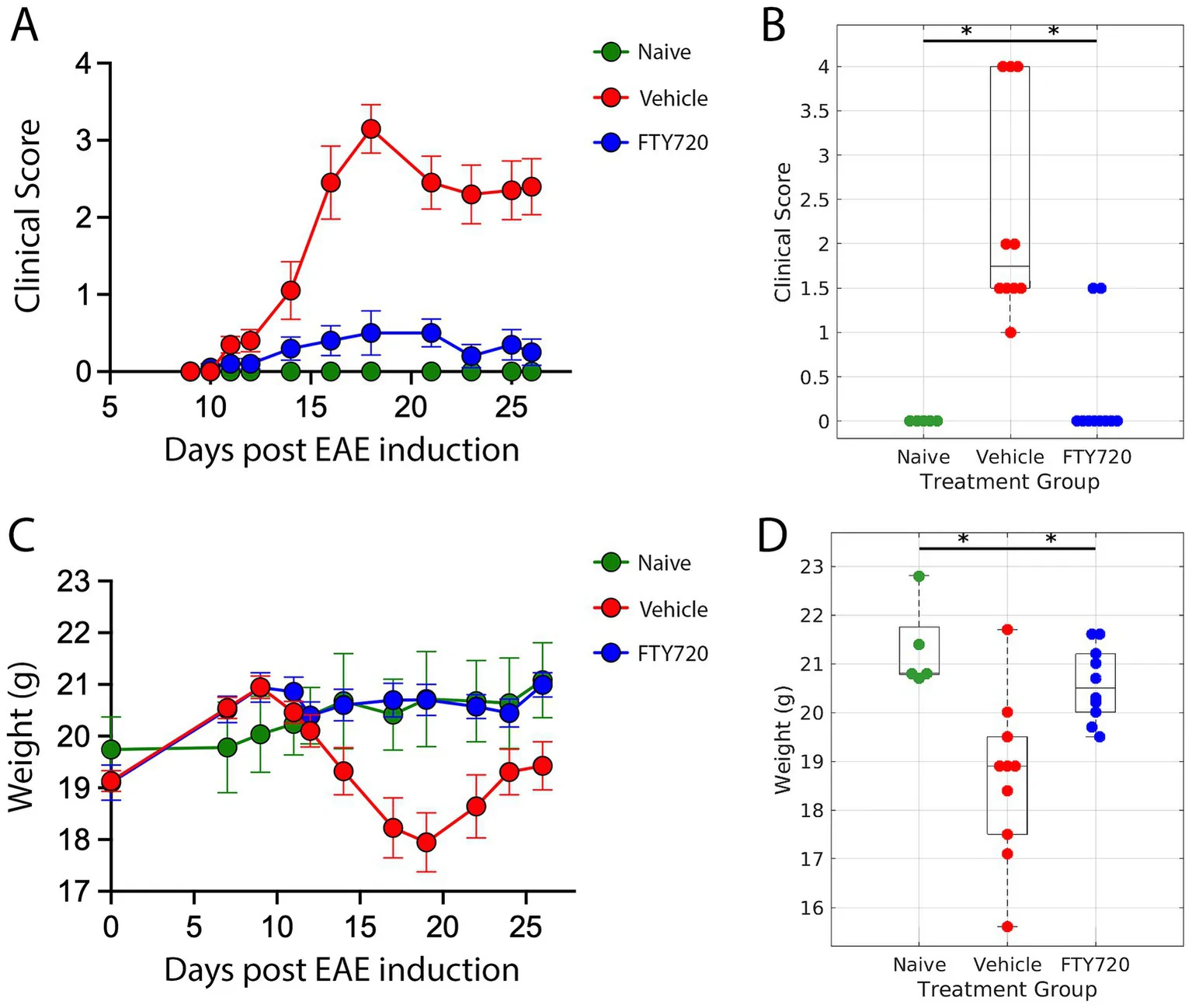
Longitudinal and terminal clinical validation of EAE disease severity and FTY720 efficacy. **(A)** Longitudinal clinical paralysis scores demonstrating the rapid onset and sustained severity of motor deficits in vehicle-treated EAE mice, compared to the sustained prevention of symptoms by FTY720. **(B)** Terminal clinical scores on day 28. **(C)** Longitudinal body weight tracking, highlighting the systemic distress and weight loss in the vehicle cohort versus the protected FTY720 and naïve groups. **(D)** Terminal body weights on day 28. Boxplots display the median and interquartile range (IQR), while error bars for longitudinal data represent the standard error of the mean (SEM), *p < 0.05.

### Histological and cellular assessment of EAE pathology

Following the macroscopic evaluations, we performed terminal, ex vivo evaluations of regional histopathology and isolated cellular populations to determine the biological basis for the MRI signals. Spatially, the vehicle group exhibited elevated histopathology scores, indicating severe demyelination and inflammatory cellular infiltration across the central nervous system (Figures 7A–D). This spatial pathology mapped closely to the localized structural deficits identified in MRI. Furthermore, this damage corresponded with robust neuroinflammation, characterized by elevated Iba1 microgliosis scores in both the brain and cervical spine (Figures 7E,F). Therapeutic intervention with FTY720 to a large extent mitigated these pathologies.

**Figure 7:**
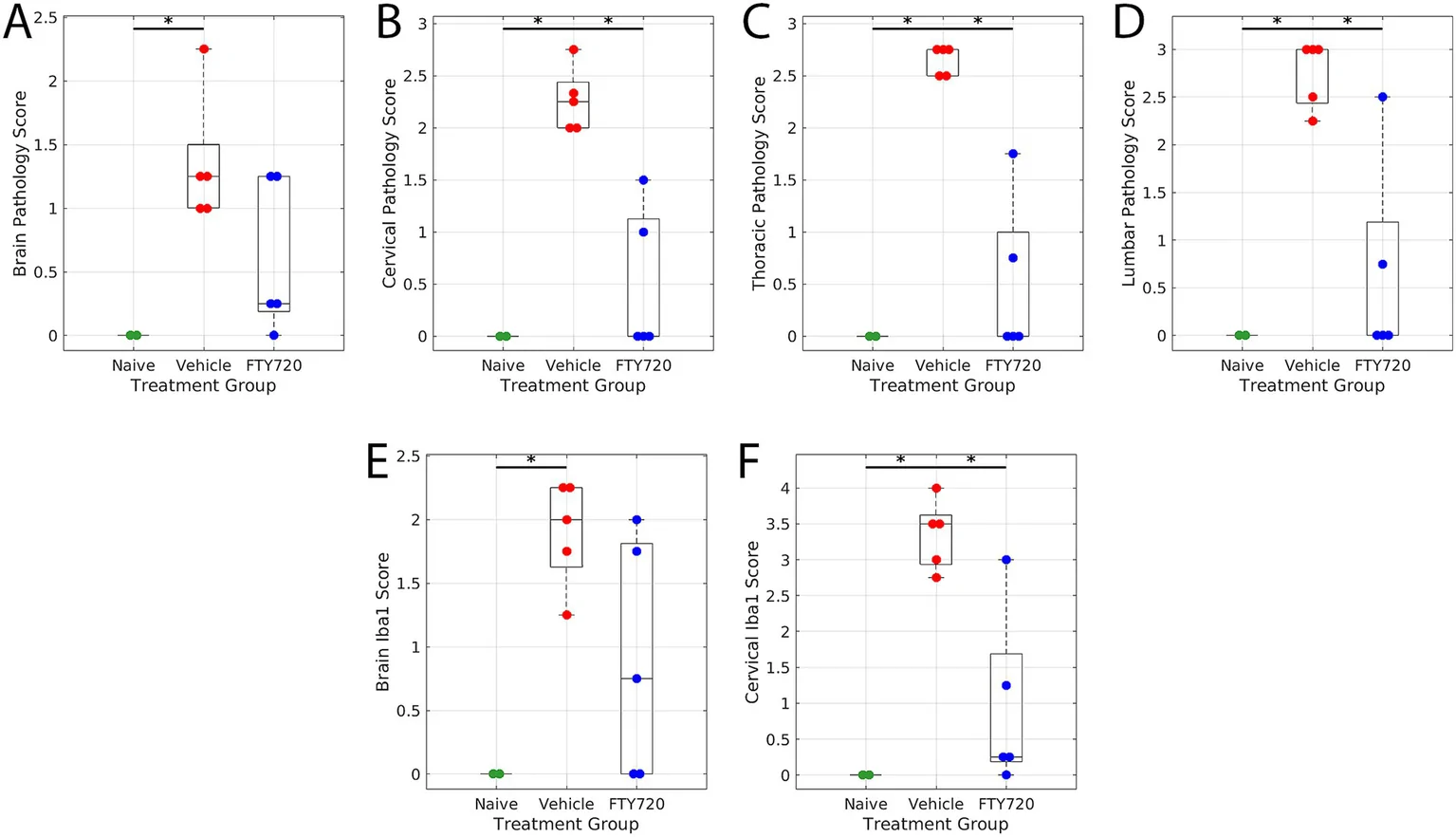
Spatial histopathology and immunohistochemical profiling at study termination. Vehicle-treated mice exhibit severe disease burden compared to naïve controls, captured by elevated H&E and LFB pathology scores in the **(A)** brain, **(B)** cervical, **(C)** thoracic, and **(D)** lumbar regions. Corresponding elevated Iba1 scores for microgliosis and macrophage infiltration are shown for the **(E)** brain and **(F)** cervical spine. FTY720 treatment rescues this spatial pathology across all evaluated anatomical levels. Boxplots display the median and interquartile range (IQR), *p < 0.05.

To quantify the driver of this inflammation, flow cytometry was utilized to profile the cellular landscape of the central nervous system (Figure 8). Representative scatter plots revealed a marked influx and distinct clustering of CD11b + myeloid cells and CD3e + T-cells in the vehicle EAE group, populations that are minimally present in the naïve central nervous system (Figures 8A,B). Quantitative analysis confirmed significant elevations in both total CNS-infiltrating T-cells and the pathogenic CD4 + T-cell subset (Figures 8C,D). Aligning directly with the preserved biophysics and clinical scores, continuous treatment with FTY720 rescued these cellular metrics, significantly reducing T-cell infiltration, decreasing Iba1 microgliosis, and preserving the local tissue architecture.

**Figure 8:**
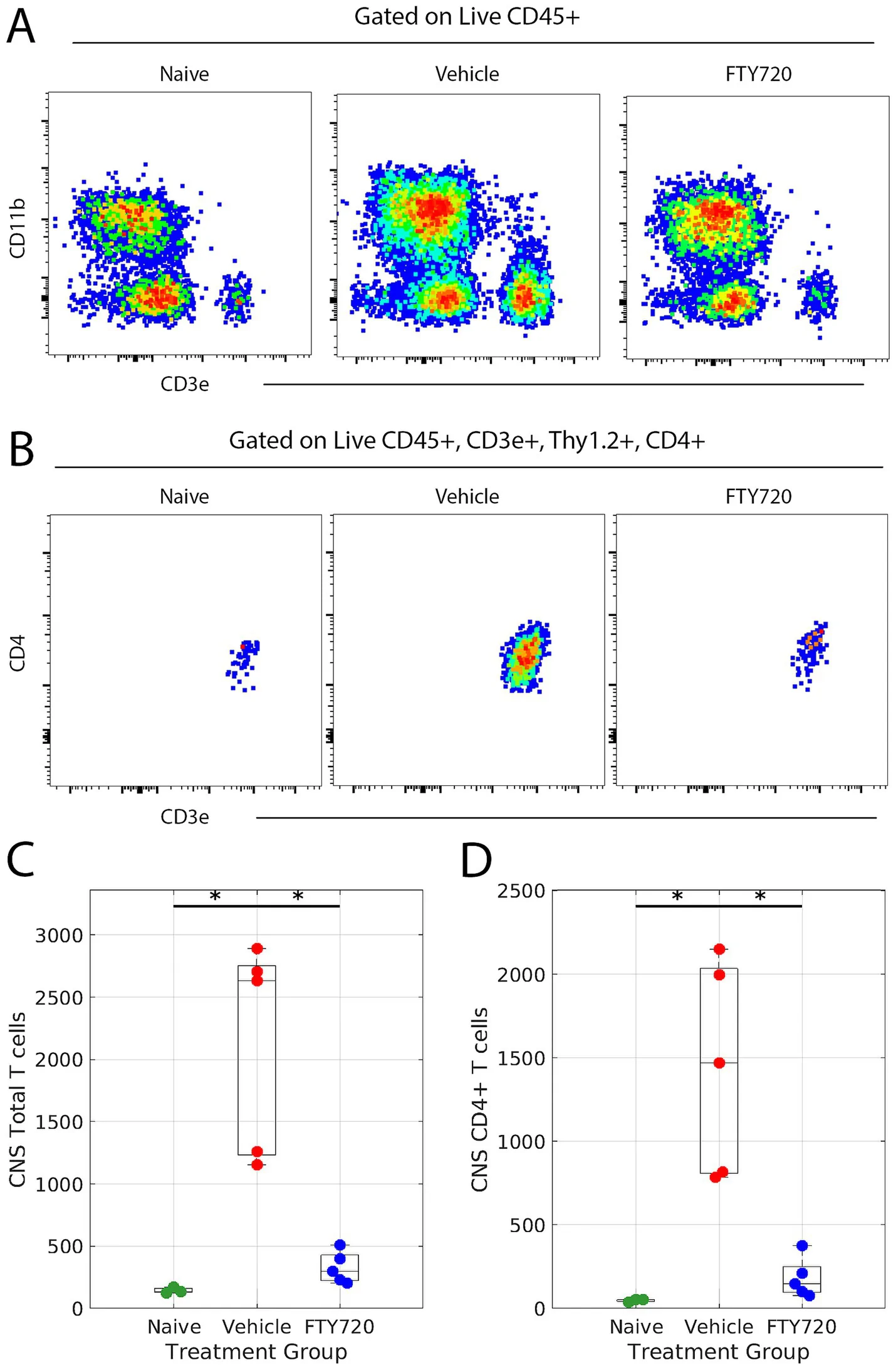
Flow cytometric quantification of CNS-infiltrating leukocyte populations. **(A)** Representative flow cytometry scatter plots showing the separation of myeloid (CD11b+) and lymphoid (CD3e+) compartments, gated on live CD45 + cells. **(B)** Representative plots detailing the marked influx of CD4 + T-cells. **(C)** Quantification of total CNS-infiltrating T-cells (CD45+/CD3e+/Thy1.2+) and **(D)** the CD4 + subset across the experimental cohorts. Vehicle EAE mice display significant elevations in total and CD4 + T-cells, which are suppressed following FTY720 administration. Boxplots display the median and interquartile range (IQR), *p < 0.05.

### Biomarker sensitivity and effect size ranking

While univariate analyses confirmed the statistical significance of the FTY720 treatment effect across modalities, we next sought to rank the absolute sensitivity of each biomarker. To quantify the magnitude of treatment-induced rescue, absolute effect sizes (Hedges’ *g*) were calculated between the vehicle and FTY720-treated EAE cohorts (Figure 5). Because variables naturally scale differently, Hedges’ *g* provides a standardized metric to directly compare the dynamic range of *in vivo* imaging against *ex vivo* pathology and clinical observations.

Within the MRI protocol (Figure 5A), lumbar GM T2 and WM FA exhibited high effect sizes, confirming them as robust, highly responsive imaging targets. Upon sub-regional analysis of the WM, the ventral FA demonstrated the highest spatial sensitivity (*g* = 2.65). This high dynamic range in the ventral columns corresponds anatomically with the primary descending motor tracts responsible for posture and hindlimb locomotion in rodents, which aligns with the clinical motor deficits observed in the model. Furthermore, the composite ratio of ventral FA to GM T2 yielded the highest overall MRI effect size, demonstrating the compounding predictive value of combining microstructural (FA) and inflammatory (T2) imaging markers.

Analysis of the biological and clinical endpoints (Figure 5B) revealed an anatomical gradient in treatment efficacy that mirrored the classical pathology of the MOG-induced EAE model. Because FTY720 mitigates active neuroinflammation, the magnitude of its protective effect was proportionally largest across the heavily diseased spinal cord (cervical, thoracic, and lumbar). Conversely, the brain pathology score yielded a lower treatment effect size, as there is less damage to rescue given the inherently spinal-cord-centric disease burden of this model. This is noticeable both in the pathologies scores and the Iba1 scores (Figure 5B). Notably, while our MRI acquisition was restricted to the lumbar cord due to hardware limitations, the histological data revealed that the thoracic region actually exhibited the highest absolute treatment effect size.

When comparing matched anatomical regions across modalities, the absolute effect sizes of the top-performing *in vivo* imaging metrics equaled or exceeded those of *ex vivo* evaluations. For example, the effect size for *in vivo* ventral FA in the lumbar spine (*g* = 2.65) was higher than the *ex vivo* histological scoring of the corresponding lumbar tissue (*g* = 2.35). Similarly, the Ventral FA and Ventral FA/GM T2 ratio exhibited greater effect sizes than the gross clinical paralysis score.

### Cross-modality correlation mapping

Having established the independent sensitivity of both radiological and biological biomarkers, we next performed cross-modality correlation analyses to determine how accurately non-invasive MRI metrics reflect underlying physiological and clinical tissue states (Figure 9A). Overall, the top-performing structural MRI metrics demonstrated robust linear relationships with gold-standard biological endpoints.

**Figure 9:**
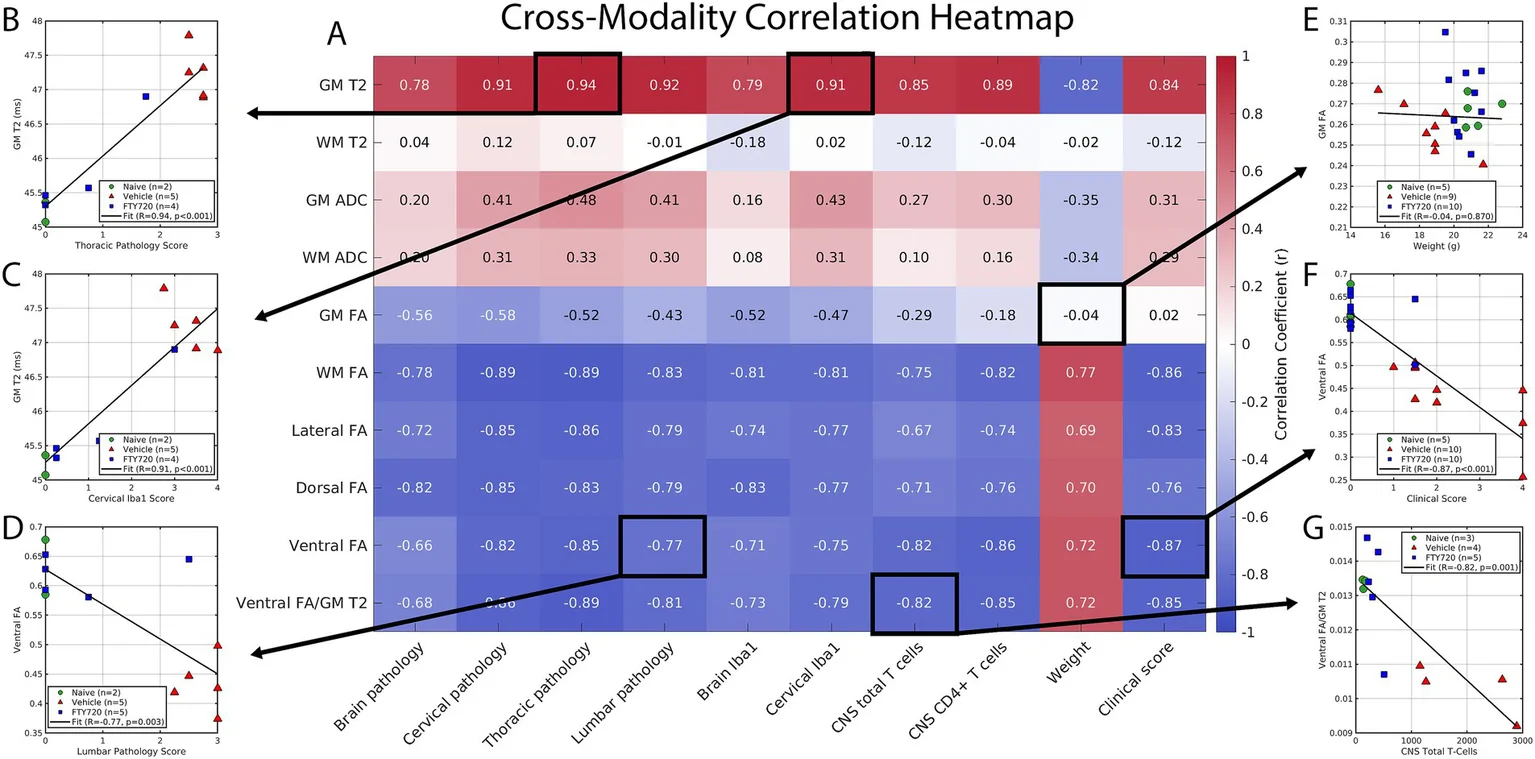
Cross-modality linear correlations between in vivo MRI and biological endpoints. **(A)** Cross-modality correlation heatmap displaying Pearson’s correlation coefficients (r) between quantitative MRI metrics (rows) and histological, cellular, and clinical endpoints (columns). Boxed cells indicate select relationships between quantitative imaging parameters and gold-standard disease outcomes, shown in the surrounding scatterplots **(B–G)**. **(B,C)** Representative strong positive correlations, demonstrating that escalating GM T2 relaxation times track with increasing histological disease severity and neuroinflammation. **(D,F,G)** Representative strong negative correlations, illustrating the inverse relationship between declining WM FA (loss of structural integrity), or the ventral FA/GM T2 composite ratio, and rising pathological scores, inflammation, and clinical motor deficits. **(E)** A scatterplot of GM FA versus body weight is provided as a benchmark, demonstrating a weak, non-significant relationship. Trendlines represent the linear best fit. Statistical strength and significance were determined using Pearson’s correlation coefficient.

Specifically, GM T2 relaxation times exhibited strong positive correlations with markers of disease severity. As GM T2 increased (indicative of worsening edema), there were tightly correlated increases in global CNS pathology scores, Iba1 microgliosis, T-cell infiltration, and clinical paralysis (example scatterplots in Figures 9B,C).

Conversely, FA across the lumbar WM and its regional sub-columns (ventral, lateral, and dorsal) displayed strong negative correlations with pathology and clinical scores (Figures 9D,F). This inverse relationship accurately reflects the physics of the metric: as neuroinflammation and clinical paralysis scores increase, the highly directional WM integrity deteriorates. Additionally, WM FA exhibited a positive correlation with systemic body weight. This is expected, as both global body weight and WM FA precipitously decline during the active EAE disease phase (vehicle) and are concurrently rescued following therapeutic intervention (FTY720). Furthermore, the composite ratio of Ventral FA to GM T2 demonstrated an inverse relationship with cellular inflammation, accurately tracking the influx of total CNS T-cells (Figure 9G).

To contextualize the strength of these relationships, metrics lacking pathological relevance in this specific model were also evaluated as benchmarks. For example, GM FA, a metric fundamentally less sensitive to demyelinating pathologies than WM FA, demonstrated a weak, non-significant relationship with body weight (Figure 9E) and clinical score, further underscoring the biological relevance of the top-ranked GM T2 and WM FA parameters.

### Multivariate network and predictive modeling

To capture the highly interconnected nature of the EAE disease state and assess the comprehensive value of the imaging protocol, we transitioned from bivariate correlations to multivariate network and predictive modeling (Figure 10). A global correlation network was first constructed to visualize the macroscopic relationships across select *in vivo* and *ex vivo* modalities simultaneously (Figure 10A). This network topology revealed that structural MRI parameters act as highly integrated central hubs with dense, significant cross-modality linkages. Among the specific imaging markers, GM T2 demonstrated the highest cumulative node strength ( ∑∣R∣=6.955 ), driven by strong linear relationships with thoracic pathology ( R=0.945 ), lumbar pathology ( R=0.920 ), and cervical pathology ( R=0.913 ). Ventral WM FA served as another dominant imaging hub in the network ( ∑∣R∣=6.306 ), displaying strong linkages to clinical score ( R=−0.871 ) and thoracic spinal cord pathology ( R=−0.850 ). The data containing all 55 connection edge coefficients and the relative node strength rankings are provided as a table in the Supplemental material.

**Figure 10:**
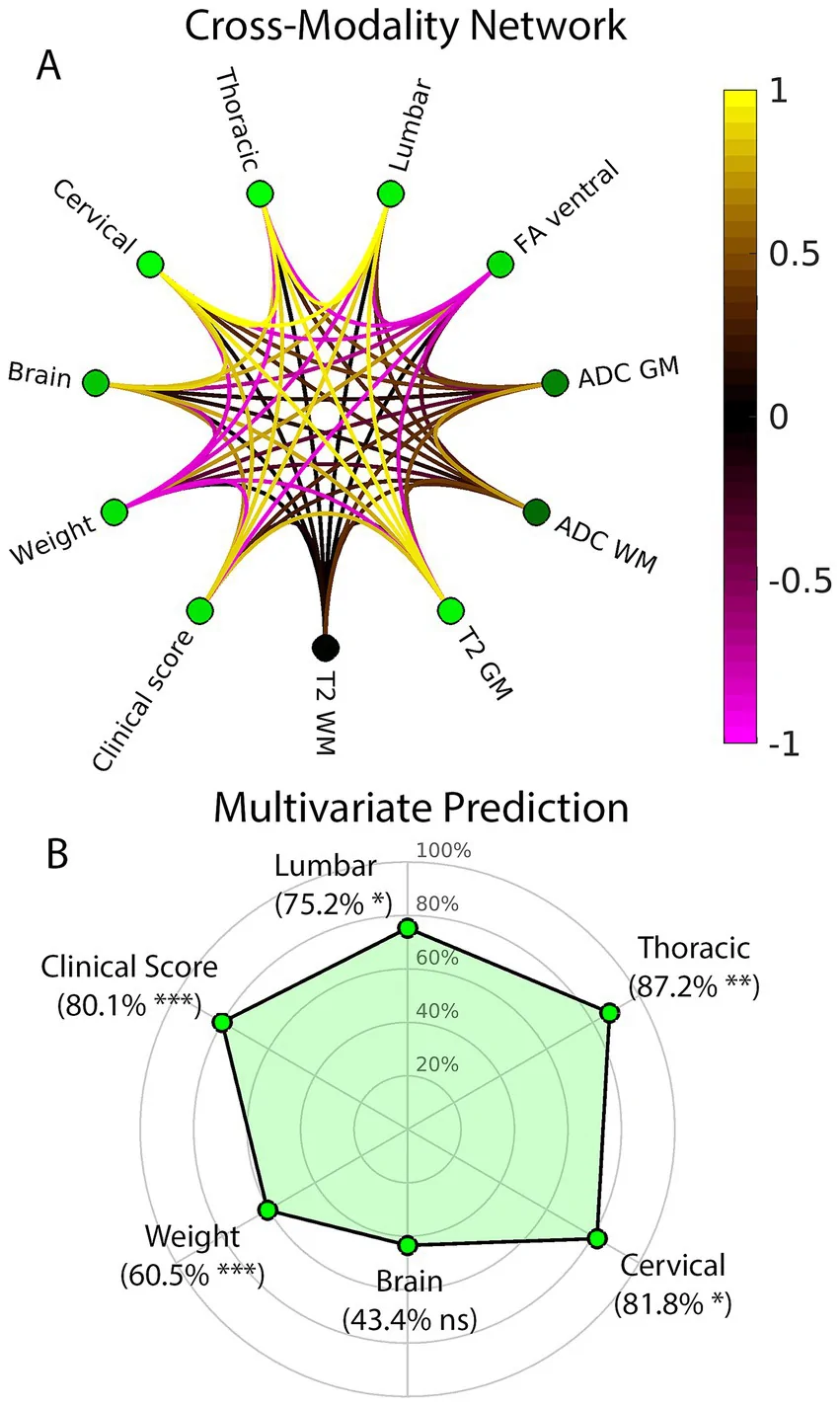
Multivariate network architecture and predictive modeling of the multiparametric MRI protocol. **(A)** A global correlation network (schemaball) visualizing the complex, interconnected relationships between select *in vivo* clinical/radiological metrics and *ex vivo* biological endpoints. Nodes representing quantitative MRI parameters act as central hubs with dense, highly significant linkages to matching cellular and systemic disease markers. Bright green circles show high cumulative correlation nodes. Bright yellow and magenta lines show high correlation links. **(B)** A multiaxial radar plot demonstrating the predictive power (adjusted *R*^2^) of a combined 5-metric MRI regression model (utilizing GM/WM T2, GM/WM ADC, and ventral FA) against gold-standard disease outcomes. The combined, non-invasive imaging panel explains a substantial proportion of the variance in regional spinal cord pathology (thoracic: 87.2%, cervical: 81.8%, lumbar: 75.2%) and clinical motor paralysis (80.1%). Conversely, the panel demonstrates lower, non-significant prediction for brain pathology (43.4%), reflecting the anatomically distinct, spinal-cord-centric burden of the EAE disease model, *p < 0.05, **p < 0.01, ***p < 0.001; *ns* = not significant.

Building upon this interconnected structure, we next sought to determine the predictive power of the imaging protocol. A multiple linear regression model was utilized to test how much of the variance in the gold-standard biological outcomes could be explained by a combined 5-metric MRI panel (GM T2, WM T2, GM ADC, WM ADC, and Ventral FA). The predictive capacity (Adjusted R^2^) of this combined radiological panel was mapped onto a multiaxial radar plot (Figure 10B).

The multivariate MRI model proved to be predictive of definitive disease endpoints. The non-invasive imaging panel accounted for a majority of the variance in spinal cord histopathology, predicting 87.2% of thoracic, 81.8% of cervical, and 75.2% of lumbar pathology scores. Furthermore, the MRI panel predicted 80.1% of the variance in the gross clinical paralysis score and 60.5% of the variance in systemic body weight. Conversely, the predictive power for brain pathology was comparatively weak and non-significant (43.4%, ns). This localized predictive disparity closely mirrors the distinct caudal-to-rostral disease progression of the EAE model, further validating that the lumbar-focused MRI protocol is highly tuned to the primary location of the pathology.

### Unsupervised phenotyping and surrogate biomarker validation

To determine if the combined multiparametric MRI protocol could blindly distinguish between healthy, diseased, and treated tissue states, we performed Principal Component Analysis (PCA) on the complete radiological dataset (Figure 11). This unsupervised dimensionality reduction successfully clustered the three experimental cohorts without any prior knowledge of their biological or clinical status.

**Figure 11:**
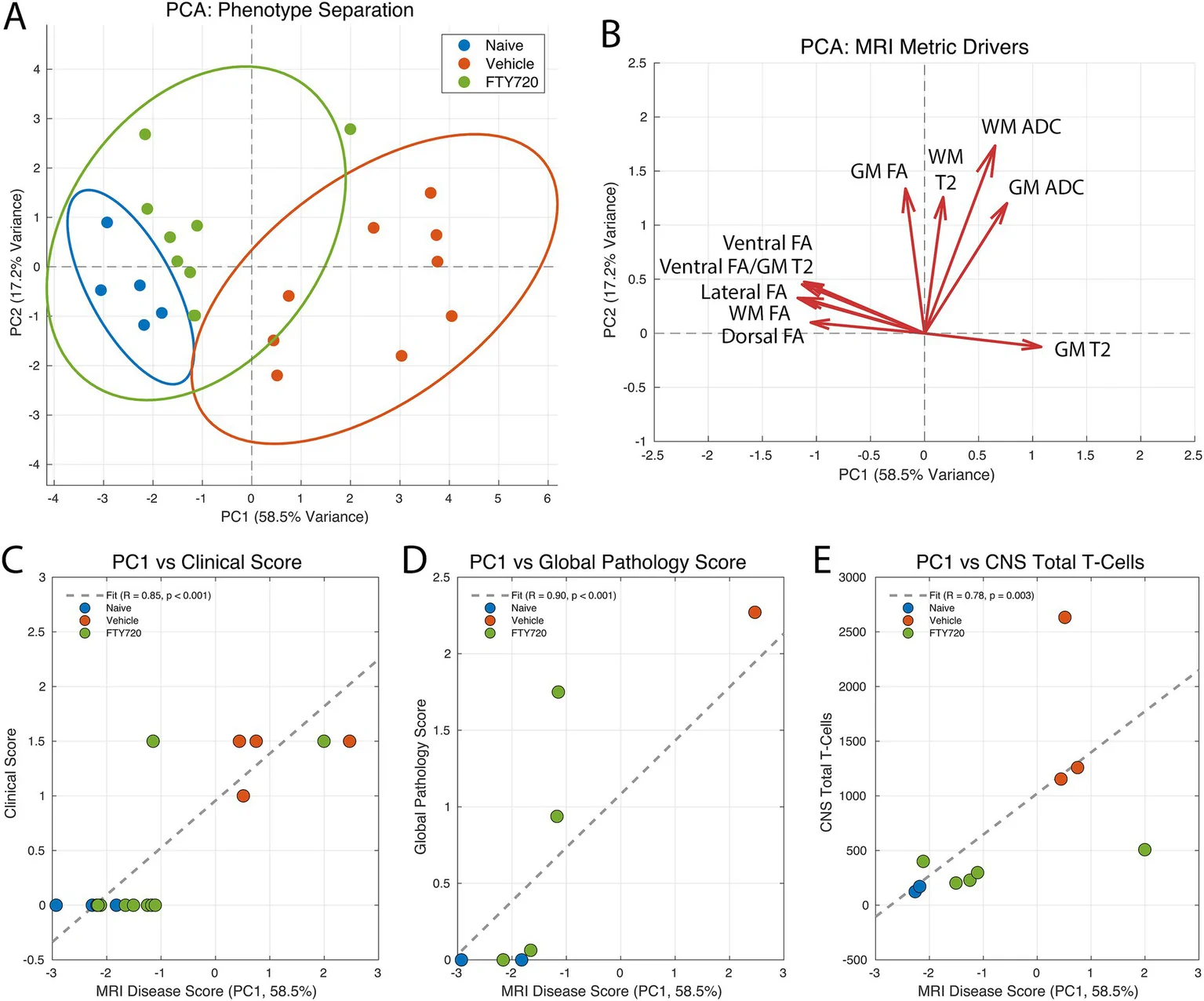
Unsupervised principal component analysis (PCA) and validation of PC1 as a surrogate biological biomarker. **(A)** A PCA biplot of all quantitative MRI metrics demonstrates unsupervised clustering of the naïve, vehicle, and FTY720-treated cohorts. **(B)** The separation along Principal Component 1 (PC1) is biologically driven, with inflammation and edema markers (GM T2) loading in opposition to microstructural integrity markers (WM FA). **(C–E)** Scatterplots demonstrating the linear correlation between the extracted radiological PC1 (MRI disease score) and matched *ex vivo* or clinical endpoints. The unsupervised PC1 score demonstrated significant correlations with **(C)** clinical paralysis score, **(D)** global histopathology score, and **(E)** total CNS T-cell infiltration. Statistical strength and significance were determined using Pearson’s correlation coefficient.

The PCA biplot (Figure 11A) revealed that the primary axis of variance (Principal Component 1, PC1) distinctly separated the severe disease state of the vehicle-treated EAE mice from the healthy naïve controls, while the FTY720-treated mice clustered in an intermediate, heavily rescued position. Evaluation of the PCA loading vectors (Figure 11B) demonstrated a biological dichotomy driving this separation: markers of edema and macroscopic tissue disruption (GM T2) strongly drove the positive axis of PC1, whereas markers of WM and microstructural integrity (regional FA metrics) strongly drove the negative axis.

Given that PC1 effectively captured the global radiological disease burden, we next evaluated its utility as a standalone, non-invasive surrogate biomarker. To do this, the individual PC1 score for each animal was extracted and linearly correlated against the gold-standard biological and clinical endpoints (Figures 11C–E). This unsupervised MRI component demonstrated strong, positive correlations with clinical paralysis score (R = 0.85, *p* < 0.001), global histopathology score (R = 0.90, *p* < 0.001), and CNS-infiltrating T-cell counts (R = 0.78, *p* < 0.01).

## Discussion

The contemporary MS therapeutic landscape is rapidly shifting (1–3). Next-generation drugs increasingly demand more nuanced *in vivo* measures of disease biology to evaluate deeper immune dampening and active remyelination (23, 24). In this study, we addressed this critical gap by developing and validating an automated, multiparametric MRI pipeline in the MOG35-55 EAE model. By benchmarking this non-invasive platform against a potent positive control (FTY720), we demonstrated that spatially targeted MRI not only provides direct, qualitative visual confirmation of *in vivo* tissue damage, but generates highly sensitive, quantitative effect sizes that accurately reflect the underlying mechanisms of the disease and the therapeutic rescue.

### Overcoming the dilution effect via spatial phenotyping

A central finding of this study is that in the evaluation of MS therapeutics, where we measure the signal is equally as important as what we measure. Standard global MRI assessments or whole-tissue histological grading frequently suffer from a mathematical dilution effect, where the signal of severe, focal lesions is averaged out by surrounding healthy tissue (10, 25). By employing an automated segmentation approach, we successfully mapped microstructural damage to precise, functionally relevant spatial locations.

The biological necessity of this spatial phenotyping is clearly reflected in our data. In the EAE model, ascending flaccid hindlimb paralysis is primarily driven by pathology within the descending motor tracts located in the ventral and ventrolateral columns of the spinal cord (15, 17). By isolating these specific ventral columns, we established a direct, spatial side-by-side correlation between the non-invasive imaging readout and the histological ground truth. The precipitous drop in FA observed in the vehicle-treated EAE mice is not merely a mathematical abstraction; it is directly underpinned by severe vacuolization, active demyelination, and dense cellular infiltrates physically breaking down the highly directional axonal tracts (16). Similarly, the localized elevations in GM T2 relaxation times mapped to areas of inflammation and gliosis. Conversely, we observed poor predictive power for brain pathology (43.4%), reflecting the inherently spinal-cord-centric disease burden of this model and further proving that averaging global CNS data mathematically dilutes the localized injury of the disease.

Crucially, the magnitude of the effect sizes generated by these localized MRI metrics translates directly into a reduction of sample sizes, thus illustrating the statistical value of overcoming the spatial dilution effect. Using a standard power analysis (⍺ = 0.05, power = 0.80) to detect a difference between vehicle and treated cohorts, the effect size observed for localized Ventral FA (g = 2.65) indicates that future studies would require approximately N = 3 to 4 mice per group. In contrast, traditional macroscopic metrics subject to the dilution effect, such as brain histopathology (g = 1.21), would require *N* = 12 mice per group to achieve the same statistical power. Furthermore, while highly targeted terminal assays like lumbar histopathology exhibit comparable statistical sensitivity (g = 2.35, N ~ 4), relying on ex vivo histology necessitates staggering separate, dedicated animal cohorts for every desired timepoint. Therefore, this non-invasive, localized MRI phenotyping not only maximizes statistical power but adheres to the 3Rs (Replacement, Reduction, and Refinement) by eliminating the need for large, multi-timepoint sacrificial cohorts.

### Evaluating the dynamic range of non-invasive imaging alongside traditional assays

While it is critical to confirm that MRI can visually track spatial changes alongside histology, the true value of a surrogate biomarker lies in its statistical sensitivity. Our biomarker sensitivity ranking demonstrated that the effect sizes of targeted MRI metrics robustly reflect the quality and strength of the imaging signal. Strikingly, the treatment effect size derived from *in vivo* ventral WM FA (*g* = 2.65) was strictly higher than the effect size of the gold-standard *ex vivo* histological scoring in the exact same lumbar tissue (*g* = 2.35).

This finding is highly consequential for preclinical study design. Standard clinical motor scores (0–5 scale) and histological grading (0–3 scale) are inherently qualitative, subject to interobserver variability, non-linear, and suffer from strict “ceiling effects” once tissue destruction reaches a macroscopic threshold (7, 26). By contrast, quantitative MRI parameters like FA and T2 provide a continuous, objective mathematical gradient. This heightened dynamic range enables targeted MRI to capture subtle, localized neuroprotective drug effects that are easily masked or missed by traditional ordinal scales. Importantly, these findings do not diminish the fundamental value of traditional endpoints. Clinical behavioral scoring remains an incredibly fast, inexpensive, and necessary tool for high-throughput, initial disease screening. Rather, our data suggest that MRI serves as a powerful, complementary modality. While macroscopic clinical scoring efficiently establishes global drug efficacy and histology provides direct cellular validation, multiparametric MRI provides the high-quality, localized tissue-level insights required to decipher exactly *how* and *where* a novel therapeutic is rescuing the central nervous system. Furthermore, the significant, independent correlations of individual parameters like Ventral FA and GM T2 with the clinical motor score suggests these metrics could be utilized as sensitive, standalone translational surrogates for early treatment response, even in the absence of complex multivariate modeling.

### The power of multivariate and composite signatures

Because MS and EAE are highly complex, multiphasic diseases, no single biophysical parameter can perfectly encapsulate the entire disease state. To provide direct confirmation that MRI measures the collective, holistic effect of disease biology and therapeutic response, we utilized large-scale cross-modality correlation measurements and unsupervised dimensionality reduction.

Our Principal Component Analysis (PCA) effectively segregated the experimental cohorts based on a fundamental biological dichotomy: markers of edema and macroscopic inflammation (GM T2) loaded in direct opposition to markers of microstructural integrity (all WM FA metrics). Furthermore, by combining these opposing biophysical metrics into a single spatially targeted composite, the Ventral FA to GM T2 ratio, we achieved the highest overall radiological effect size. The subsequent extraction of the PCI score as a standalone MRI disease score yielded strong correlations with clinical disability, global histopathology, and total CNS T-cell infiltration. Together, these multivariate signatures confirm that advanced MRI can reliably capture the complex interplay of inflammation and neurodegeneration without the need to sacrifice the animal.

### Translational and technical considerations of MRI metrics

While our data highlights the sensitivity of GM T2 relaxation times in this model, we recognize a divergence from clinical MS presentation, where WM T2 hyperintensity remains the diagnostic hallmark for demyelinating plaques. In our analysis, WM T2 exhibited a surprisingly low sensitivity and treatment effect size (Figure 5A). This discrepancy is likely driven by a combination of technical imaging limitations and the biological characteristics of the model.

Technically, the accurate quantification of WM T2 in the murine spinal cord is inherently susceptible to partial volume effects during image segmentation. While DTI FA maps provide high-contrast boundaries between the anisotropic WM and adjacent tissues, structural T2 maps exhibit much lower contrast between the thin WM tracts, the central GM, and the surrounding cerebrospinal fluid (CSF). Consequently, even minor spatial misalignments can lead to the inadvertent inclusion of adjacent CSF voxels. Because CSF possesses much higher T2 relaxation times, this contamination can skew the average WM T2 measurements and potentially mask subtle, treatment-induced pathological changes.

From a biological perspective, severe murine MOG35-55 EAE features massive, diffuse cellular infiltration into the central GM rather than the highly focal white matter plaques seen in human MS. Mechanistically, this inflammatory influx and blood-spinal cord barrier disruption drive localized vasogenic edema, expanding the pool of free, mobile water molecules and physically prolonging quantitative T2 relaxation times. Conversely, white matter FA is governed by structural boundaries. While intact, parallel axons and hydrophobic myelin sheaths restrict water movement to a single primary axis, inflammatory demyelination and axonal fragmentation dismantle these physical barriers, allowing water to diffuse randomly (isotropically) and causing a precipitous drop in FA. Together, these biophysical traits position T2 as a marker for neuroinflammatory fluid shifts, and FA as a surrogate for microstructural tracking.

Conversely, high-contrast metrics of microstructural directionality (WM FA) currently remain an optimal, highly accessible surrogate for tracking demyelination in the WM. However, future preclinical pipelines may further benefit from advanced, myelin-specific MR techniques to overcome these spatial limitations. For instance, Myelin Water Imaging has been highly validated for isolating demyelinating pathology in both the brain and spinal cord (27). Moreover, the implementation of Ultrashort Echo Time (UTE) MRI allows for a more direct detection of the rapidly decaying signal from the myelin lipid bilayer itself (28, 29). Integrating these advanced modalities into longitudinal pipelines could reduce the partial volume confounds of CSF and provide an improved quantification of remyelination.

Beyond the model-specific constraints of murine neuroinflammation, fundamental translational boundaries should be considered when scaling this pipeline to clinical settings. High-resolution column parcellation in this study is heavily dependent on high-field MRI combined with a specialized cryogenic radiofrequency coil, achieving a native in-plane resolution of 65 μm. In clinical practices, achieving a similar resolution within the human spinal cord at 1.5 T or 3 T remains physically constrained by signal-to-noise limitations and motion artifacts. Furthermore, a clear divergence exists between preclinical behavioral tracking and human clinical endpoints. The 0–5 EAE motor scale is inherently restricted to crude, ascending hindlimb paralysis, whereas clinical disability tracking (e.g., the Expanded Disability Status Scale) evaluates a broader network: visual acuity, cognitive processing, and fine motor coordination. Ultimately, this framework serves as a preclinical surrogate for screening novel therapeutics rather than a formula for clinical trials.

### Limitations and future directions

While this study successfully validated the spatial and biological fidelity of the multiparametric MRI protocol, it was intentionally designed as a cross-sectional evaluation paired with terminal *ex vivo* ground truths at an advanced timepoint (Day 28 post-induction). This cross-sectional framework is an acknowledged limitation, as it restricts our capacity to map the continuous temporal evolution of neurodegeneration and therapeutic rescue over time. However, this design was chosen as an essential baseline validation phase. Because our primary biological gold standards (multi-level histopathology and flow cytometry) are inherently destructive terminal assays that require incompatible tissue pathways, a true multi-timepoint longitudinal validation against tissue-level metrics would necessitate large, independent animal cohorts sacrificed across staggered timepoints. Establishing that localized *in vivo* biophysics match destructive tissue readouts at a static endpoint was a necessary prerequisite before deploying future longitudinal studies.

Adding multiple imaging timepoints in future studies can enhance the utility of this platform, allowing researchers to capture the temporal dynamics of disease evolution and the real-time dynamic response of novel therapeutics (30–32). Furthermore, incorporating baseline MRI scans prior to disease induction offers critical methodological advantages. Baseline imaging allows for the rigorous counterbalancing of experimental cohorts, ensuring that subtle, pre-existing microstructural variances do not confound treatment outcomes. Additionally, normalizing longitudinal data to an animal’s own internal baseline mathematically removes inherent biological variance, revealing the true, isolated effect of the drug rather than artifacts of mismanaged animal grouping.

Generating highly powered, continuous longitudinal data enables the calculation of the Area Under the Curve (AUC) for MRI metrics over time. Utilizing AUC can enhance the statistical separation between experimental groups, maximizing the detectable magnitude of a drug’s effect (or lack thereof) (33, 34). Transitioning this validated spatial protocol into fully longitudinal efficacy trials can provide nuanced insights into the mechanisms of future immune-dampening and remyelinating interventions. Additionally, while our unsupervised multivariate framework demonstrates robust natural clustering within this cohort, its generalizability is currently limited by a lack of validation in independent datasets. Future studies or testing against distinct classes of experimental therapeutics will be essential to confirm the reproducibility of these specific principal component loading weights.

A secondary limitation of this study was the restriction of the MRI field of view strictly to the lumbar spinal cord, which was necessitated by the range limitations of the MRI cryoprobe. However, our ex vivo biomarker sensitivity ranking demonstrated that the treatment effect size was highest within the thoracic spinal cord. Consequently, our lumbar-restricted protocol demonstrates a robust treatment effect but inherently serves as a conservative underestimation of the global therapeutic rescue across the central nervous system. This spatial restriction limits the direct generalizability of these specific biomarkers to descending lumbar motor tracts and hindlimb scores, rather than whole-cord or brain pathology. Therefore, if future studies employ larger volumetric coils or multi-station sequential imaging to capture these severe thoracic lesions, the generalizability, predictive effect sizes, and dynamic range of this non-invasive MRI protocol can be further enhanced.

## Conclusion

The development of highly targeted, disease-modifying therapies for multiple sclerosis requires equally sophisticated preclinical evaluation platforms. We demonstrate that an automated, spatially precise, multiparametric MRI pipeline mirrors the complex biological and clinical trajectory of the EAE model. By targeting the specific microstructural tracts responsible for clinical disability, non-invasive *in vivo* imaging yields statistical effect sizes that meet or exceed traditional invasive gold standards. Ultimately, this approach serves as a highly sensitive, quantitatively objective surrogate biomarker that enhances the rigorous evaluation of MS therapeutics while advancing the ethical reduction and refinement of animal use.

## Data Availability

The original contributions presented in the study are included in the article/Supplementary material, further inquiries can be directed to the corresponding authors.

## Supplementary material

SUPPLEMENTARY TABLE 1 Per-animal source data across in vivo MRI, in vivo clinical and ex vivo pathology endpoints.

SUPPLEMENTARY TABLE 2 Longitudinal body weight per animal.

SUPPLEMENTARY TABLE 3 Per-animal counts of Total CNS T-cells and CD4+ T-cells.

SUPPLEMENTARY TABLE 4 Connection (edge) coefficients of the correlation network (55 pairwise Pearson correlations), and cumulative node strength (Σ|R|) used to size/color the schemaball nodes.

SUPPLEMENTARY TABLE 5 Parametric versus non-parametric concordance: normality assessment, Pearson versus Spearman correlations, and t-test versus Mann–Whitney U.

SUPPLEMENTARY TABLE 6 Exact sample size (n) per group for every analyzed variable, and primary two-group comparisons (Vehicle/EAE vs FTY720) with raw and multiplicity-corrected p-values, effect size, and achieved power.

SUPPLEMENTARY TABLE 7 Sensitivity power analysis - minimum detectable effect size (MDES) for the realized group sizes.

SUPPLEMENTARY TABLE 8 Multicollinearity of the multivariate MRI regression panel (variance inflation factors).

## Glossary

AAALAC: American Association for the Accreditation of Laboratory Animal Care
AD: Axial diffusivity
ADC: Apparent diffusion coefficient
ANTs: Advanced Normalization Tools
AUC: Area under the curve
CFA: Complete Freund’s Adjuvant
CNS: Central nervous system
CSF: Cerebrospinal fluid
DTI: Diffusion tensor imaging
DWI: Diffusion-weighted images
EAE: Experimental autoimmune encephalomyelitis
FA: Fractional anisotropy
FOV: Field of View
FTY720: Fingolimod hydrochloride
GM: Gray matter
H&E: Hematoxylin and eosin
Iba1: Ionized calcium-binding adapter molecule 1
IHC: Immunohistochemistry
LFB: Luxol fast blue
MOG35-55: Myelin oligodendrocyte glycoprotein
MRI: Magnetic resonance imaging
MS: Multiple sclerosis
MSME: Multi-slice multi-echo
PBS: Phosphate-buffered saline
PC1: Principal Component 1
PCA: Principal Component Analysis
SNR: Signal-to-noise ratio
TE: Echo time
TR: Repetition time
WM: White matter
